# Augmentation of Ca^2+^ signaling in astrocytic endfeet in the latent phase of temporal lobe epilepsy

**DOI:** 10.3389/fncel.2015.00049

**Published:** 2015-02-25

**Authors:** Karolina Szokol, Kjell Heuser, Wannan Tang, Vidar Jensen, Rune Enger, Peter Bedner, Christian Steinhäuser, Erik Taubøll, Ole Petter Ottersen, Erlend A. Nagelhus

**Affiliations:** ^1^Department of Neurology, Oslo University HospitalOslo, Norway; ^2^Centre for Molecular Medicine Norway, The Nordic EMBL Partnership, University of OsloOslo, Norway; ^3^Letten Centre and GliaLab, Department of Physiology, Institute of Basic Medical Sciences, University of OsloOslo, Norway; ^4^Institute of Cellular Neurosciences, University of BonnBonn, Germany; ^5^University President’s Office, University of OsloOslo, Norway

**Keywords:** astrocytes, GCaMP, dystrophin, glia, kainate, perivascular

## Abstract

Astrocytic endfeet are specialized cell compartments whose important homeostatic roles depend on their enrichment of water and ion channels anchored by the dystrophin associated protein complex (DAPC). This protein complex is known to disassemble in patients with mesial temporal lobe epilepsy and in the latent phase of experimental epilepsies. The mechanistic underpinning of this disassembly is an obvious target of future therapies, but remains unresolved. Here we show in a kainate model of temporal lobe epilepsy that astrocytic endfeet display an enhanced stimulation-evoked Ca^2+^ signal that outlast the Ca^2+^ signal in the cell bodies. While the amplitude of this Ca^2+^ signal is reduced following group I/II metabotropic receptor (mGluR) blockade, the duration is sustained. Based on previous studies it has been hypothesized that the molecular disassembly in astrocytic endfeet is caused by dystrophin cleavage mediated by Ca^2+^ dependent proteases. Using a newly developed genetically encoded Ca^2+^ sensor, the present study bolsters this hypothesis by demonstrating long-lasting, enhanced stimulation-evoked Ca^2+^ signals in astrocytic endfeet.

## Introduction

Evidence is accruing that perivascular astrocytic endfeet are highly specialized cell compartments in terms of molecular organization and functional roles (Nagelhus and Ottersen, 2013). Many of the unique features of these processes can be explained by their expression of the brain dystrophin DP71 which orchestrates a molecular assembly that includes the water channel aquaporin-4 (AQP4; Frigeri et al., 2001; Neely et al., 2001; Enger et al., 2012; Waite et al., 2012). The endfoot complement of AQP4 determines the rate by which water accumulates in brain in conditions favoring the development of brain edema (Vajda et al., 2002; Amiry-Moghaddam et al., 2003; Haj-Yasein et al., 2011b). The endfeet are also enriched in the inwardly rectifying K^+^ channel Kir4.1 (Nagelhus et al., 1999; Higashi et al., 2001). This channel is thought to mediate K^+^ siphoning in the retina (Kofuji et al., 2000) and contributes to K^+^ spatial buffering in the CNS at large (Chever et al., 2010; Haj-Yasein et al., 2011a). The unique features of the astrocytic endfeet imply that astrocytes are highly polarized cells, biochemically as well as functionally.

It was recently found that loss of astrocyte polarization is common to several neurological conditions. The endfoot pool of AQP4 drops abruptly after an ischemic insult (Frydenlund et al., 2006; Steiner et al., 2012), and is also strongly reduced in models of Alzheimer’s disease (Yang et al., 2011) and traumatic brain injury (Ren et al., 2013). Similarly, loss of astrocyte polarization—with reductions in AQP4 as well as Kir4.1—has been described in the hippocampus of patients with temporal lobe epilepsy (Schröder et al., 2000; Eid et al., 2005; Heuser et al., 2012). These changes are reproduced in experimental models of epilepsy, including the kainate model (Lee et al., 2012; Alvestad et al., 2013). The loss of Kir4.1 in particular is likely to be pathophysiologically relevant, as glial-conditional Kir4.1 knockout animals display deficient K^+^ spatial buffering and severe epilepsy (Chever et al., 2010; Haj-Yasein et al., 2011a). Disassembly of endfoot protein complexes emerges as one of several mechanisms whereby astroglia may contribute to hyperexcitability and epileptogenesis (Binder et al., 2012; Binder and Carson, 2013; Crunelli et al., 2014).

The mechanisms underlying the loss of astrocyte polarization in epilepsy have not been resolved. One possible mechanism is that an early injury causes Ca^2+^ accumulation in endfeet, leading to proteolytic cleavage of the dystrophin associated protein complex (DAPC) at these sites. Such a mechanism is plausible, as astrocytes activated by injury contain calpain (Shields et al., 2000)—a protease that shows affinity to dystrophin and that is activated by Ca^2+^ (Yoshida et al., 1992).

This hypothesis cannot be tested by conventional Ca^2+^ imaging, as bulk-loaded synthetic Ca^2+^ dyes mainly reveal Ca^2+^ signals at the level of the cell bodies (Reeves et al., 2011). Here we use an approach that allows Ca^2+^ signals to be monitored in the fine astrocytic processes, including the perivascular endfeet. Specifically, we employed recombinant adeno-associated virus (rAAV) gene delivery of the genetically encoded Ca^2+^ indicator GCaMP5E (Akerboom et al., 2012) to hippocampal astrocytes in a mouse model of temporal lobe epilepsy. Two-photon Ca^2+^ imaging of acute hippocampal slices obtained in the epilepsy latent phase revealed elevated stimulation-evoked astrocytic Ca^2+^ signals, both in endfeet and in astrocytic cell bodies. Indeed, the Ca^2+^ signals in endfeet outlasted those in cell bodies. The present data point to endfoot Ca^2+^ signaling as a possible mechanism underpinning the loss of astrocyte polarization in epilepsy.

## Material and methods

### Animals

Male C57BL/6N mice of 2–4 months of age (Charles River) were used for all experiments. All procedures were approved by the animal use and care committee of the Institute of Basic Medical Sciences, University of Oslo, and the Centre for Comparative Medicine, Oslo University Hospital.

### Plasmid constructs

The plasmid constructs were generated as described in a separate paper (Tang et al., 2015). In brief, the GCaMP5E DNA sequence was directly taken out from the expression vector pRGCAMP5E (Akerboom et al., 2012) by restriction digest with BamHI and HindIII, and subcloned into the rAAV vector pAAV-6P-SEWB (Shevtsova et al., 2005) with the human *SYNAPSIN-1* (*SYN*) promoter to generate the construct of pAAV-*SYN*-GCaMP5E. The human *GFAP* promoter (Hirrlinger et al., 2009) was then inserted with MluI and BamHI into the pAAV-*SYN*-GCaMP5E vector resulting in the pAAV-*GFAP*-GCaMP5E construct.

### Viral transduction

rAAVs serotype 1 and 2 were generated as described (Tang et al., 2009), and purified by AVB Sepharose affinity chromatography (Smith et al., 2009). For the virus preparation, the genomic titer was determined by Real-Time PCR (~1.0 × 10^12^ viral genomes (vg)/ml, TaqMan Assay, Applied Biosystems). For virus infection, adult mice were deeply anesthetized with a mixture of zolazepam (188 mg/kg body weight), tiletamine (188 mg/kg body weight), xylazine (4.5 mg/kg body weight) and fentanyl (26 µg/kg body weight) before viruses were stereotactically injected (Shevtsova et al., 2005) into both hippocampi. Coordinates relative to Bregma were: anteroposterior −2.0 mm, lateral ±1.5 mm, depth 1.5 mm. During each injection, 0.3 µl of purified rAAV (~1.0 × 10^12^ vg/ml) was delivered.

### Intracortical kainate injection model for mesial TLE

We used deep cortical (juxtahippocampal) kainate injection to elicit an initial status epilepticus (SE). Using this approach, more than 90% of injected animals developed recurrent behavioral seizures after a 5–8 day long latent period. For kainate injections, mice were anesthetized with a mixture of medetomidine (0.3 mg/kg, i.p.) and ketamine (40 mg/kg, i.p.) and kept on a heating blanket. A small craniotomy was performed in a stereotactic frame and kainate (50 nl; 20 mM; Tocris) was injected by a Hamilton pipette (Hamilton Company, NV) at a depth of 1.7 mm at the following coordinates relative to Bregma: anteroposterior −2 mm, lateral +1.5 mm (right). Anesthesia was stopped with atipamezol (300 mg/kg, i.p.) and SE was observed either clinically or by telemetric EEG recording and video monitoring. The animal model has been described in detail in a separate paper (Bedner et al., 2015). The non-injected side served as control for the kainate injected side.

### Immunohistochemistry and confocal imaging

Virus transduced mice were anesthetized with ~4% isoflurane and intracardially perfused with 1 × phosphate buffered saline (PBS; 137 mM NaCl, 2.7 mM KCl, 4.3 mM Na_2_HPO_4_/2H_2_O, 1.4 mM KH_2_PO_4_, pH 7.4, all from Sigma-Aldrich) and 4% paraformaldehyde (PFA, Merck) in PBS prior to decapitation. Brains were removed and fixed in ice-cold 4% PFA/PBS for 2 h, embedded in 2.5% Agarose (Invitrogen) in PBS and sliced on a Vibratome (Leica) into 70 µm sections. Immunostaining was performed with polyclonal rabbit anti-GFP (1:3000, Abcam, #ab6556), chicken anti-GFAP (1:1000, Covance, #PCK-591P), rat anti-CD31 (1:200, BD Biosciences, #550274) and FITC-coupled anti-rabbit, Cy3-coupled anti-chicken, Cy5-coupled anti-rat secondary antibodies (1:200, Jackson Immuno Research, #711095152, #703165155 and #712175153 respectively). Confocal images were acquired on a Zeiss LSM5 PASCAL confocal laser scanning microscopy with 63x/1.4NA oil-immersion objective, equipped with an Argon laser (457, 476, 488, 514 nm) and a Helium Neon laser (543 nm, Carl Zeiss).

### Electrophysiology and two-photon Ca^2+^ imaging

Experiments were performed on hippocampal slices prepared 3–4 weeks after injection of rAAV-*GFAP*-GCaMP5E and 1, 3 and 7 days after juxtahippocampal, cortical kainate injections. The animals were sacrificed with an overdose of desflurane (Baxter), and brains were removed and cooled in artificial cerebrospinal fluid (ACSF, 0–4°C, bubbled with 95% O_2_/5% CO_2_, pH 7.4) containing (in mM): 124 NaCl, 2 KCl, 1.25 KH_2_PO_4_, 2 MgSO_4_, 1 CaCl_2_, 26 NaHCO_3_ and 12 glucose. Transverse slices (400 µm) were cut from the dorsal portion of each hippocampus with a Vibratome slicer (Leica) and placed in a humidified interface chamber at 30 ± 1°C and perfused with ACSF containing 2 mM CaCl_2_. In some experiments the group I/II mGluR antagonist α-methyl-4-carboxyphenylglycine (MCPG; 1 mM, Tocris) or the mGluR5 selective antagonist 2-methyl-6-phenylethynyl pyridine hydrochloride (MPEP; 100 µM, Tocris) were added to the ACSF. Two glass electrodes filled with ACSF and positioned 200–300 µm away from each other in CA1 *stratum radiatum* served as stimulation and recording electrodes, respectively. Orthodromic synaptic stimulations at 20 Hz for 10 s were delivered and excitatory postsynaptic potentials (fEPSPs) were monitored. Neuronal stimulation-evoked (simultaneous recording while electrical stimulations) astrocytic GCaMP5 fluorescence signals were recorded by a two-photon laser scanning microscope (model “Ultima”, Prairie Technologies), as described previously (Tang et al., 2015). Astrocytic Ca^2+^ responses on the kainate injected side were compared with those on the non-injected (control) side. Images were recorded with a model “XLPLN 25 × WMP” 1.05NA, water-immersion objective (Olympus, Japan) at 900–910 nm laser pulses. The laser was a model “Chameleon Vision II” (Coherent, Santa Clara, CA). The recording was done either with 1 Hz or 4 Hz frame rate, the images were 512 × 512 px or 256 × 256 px, respectively.

### Imaging analysis

Time-series of fluorescence images were first imported into Fiji ImageJ (Fiji), and regions of interest (ROIs) were manually selected based on morphology. Astrocytic cell bodies were identified by their projecting branches and endfeet by their characteristic circular pattern around transversely cut vessels and elongated, linear appearance along obliquely cut vessels. ROIs over processes were chosen at least 5 µm away from the perimeter of the soma. The relative change in fluorescence (ΔF/F) in each ROI, the individual traces and the histograms were all calculated and plotted by MATLAB (R2011b, MathWorks, Inc.) with custom written scripts. Standard deviation (SD) images were generated from time-lapse image recordings by Fiji ImageJ.

### Statistical analysis

Statistical analyses were performed using Prism (Version 6.0b for Mac OSX, GraphPad Software). One-way ANOVA with Tukey multiple comparisons test was used for comparison of GCaMP5E fluorescence changes in astrocytic somata, processes and endfeet following stimulation of Schaffer collaterals/commissural fibers. Paired *t*-test was used for comparison before and after wash-in with MCPG and MPEP. *P* < 0.05 was considered statistically significant.

## Results

### Viral transduction yielded expression of the Ca^2+^ indicator GCaMP5E in adult mouse hippocampal astrocytes

**Figure 1:**
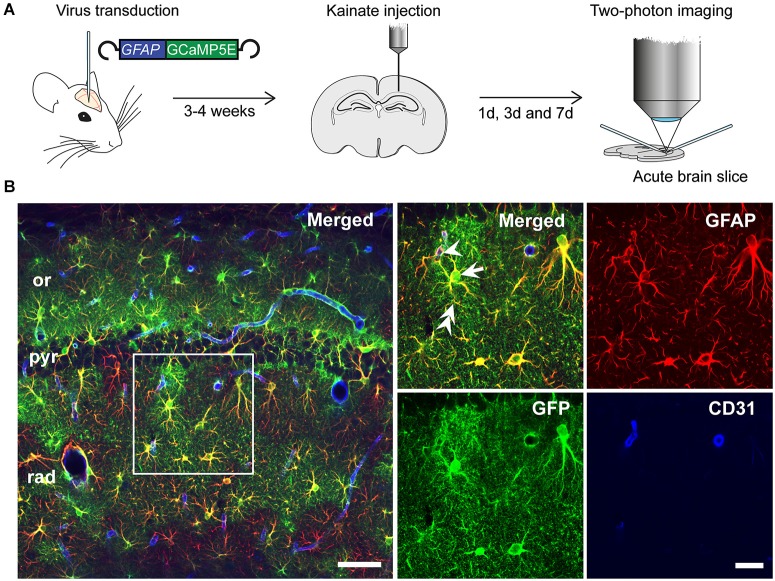
**Experimental design. (A)** rAAV-*GFAP*-GCaMP5E virus was injected into both hippocampi 2–3 weeks prior to unilateral, intracortical kainate injection. Acute hippocampal slices were prepared and imaged at 1, 3 and 7 days post kainate injection. **(B)** Immunofluorescence with green fluorescent protein (GFP) antibodies (green) showed robust GGaMP5E expression in GFAP immunopositive (red) astrocytes. The vascular endothelium was labeled with CD31 antibodies (blue). Arrow: astrocyte soma, double arrow: astrocyte process, arrowhead: endfoot. or, *stratum oriens*; pyr, *stratum pyramidale*; rad, *stratum radiatum*. Scale bars, 50 µm and 20 µm (boxed motif expanded in inset).

Injection of the rAAV-GFAP-GCaMP5E construct into the hippocampus yielded robust and selective GCaMP5E expression in hippocampal astrocytes, as revealed by immunolabeling with antibodies against green fluorescent protein (GFP) and glial fibrillary acidic protein (GFAP; Figures 1A,B). Notably, GCaMP5E was expressed within all astrocytic compartments, including the fine astrocytic processes and endfeet adjacent to CD31-immunopositive blood vessels (Figure 1B).

### Stimulation induced Ca^2+^ signals within astrocytic somata, processes and endfeet are enhanced in the latent phase of epilepsy

**Figure 2:**
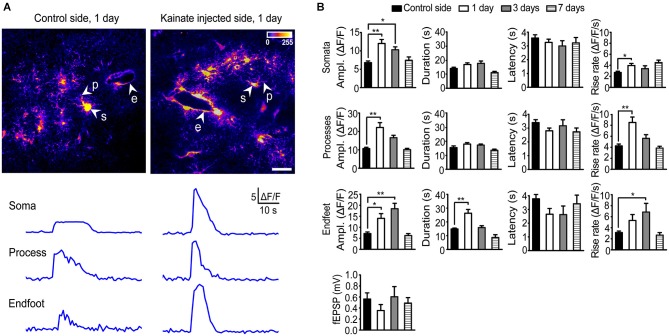
**Two-photon imaging of stimulation-evoked astrocytic Ca^2+^ signals in adult mouse hippocampal slices 1, 3 and 7 days after intracortical kainate injection. (A)** Standard deviation (SD) images of GCaMP5E fluorescence intensities from 1 Hz time-lapse recording 1 day after kainate injection. GCaMP5E fluorescent astrocytic somata (s), processes (p), and endfeet (e) are indicated. The fluorescence traces from the astrocytic compartments indicated in the images are shown below. Scale bar, 20 µm.** (B)** Amplitudes, duration, latency and rise rate of Schaffer collateral/commissural fiber (Scc) stimulation (20 Hz, 10 s) evoked GCaMP5E fluorescence transients in astrocytic somata, processes and endfeet in control (non-injected side; 20 mice, 22 slices), 1 day (11 mice, 16 slices), 3 days (3 mice, 6 slices) and 7 days (6 mice, 9 slices) after kainate injection. Lower panel shows average fEPSP for the different time points. Values are mean ± s.e.m. Asterisk, *P* < 0.05. Double asterisk, *P* < 0.0001.

Stimulation (20 Hz, 10 s) of Schaffer collateral/commissural fibers (Scc) in acute hippocampal slices from the control side of rAAV-*GFAP*-GCaMP5E-transduced animals 1 day after intracortical kainate injection elicited brisk Ca^2+^ signals in the majority of *stratum radiatum* astrocytes (Figure 2A), as reported for slices from healthy adult mice (Tang et al., 2015). Compared to the control side, the amplitudes of stimulation evoked Ca^2+^ signals in the kainate injected side were significantly increased in all astrocytic compartments 1 day post injection (Figures 2A,B; soma contralateral 6.8 ± 0.4 vs. ipsilateral 12.0 ± 1.1, *P* < 0.0001, *n* = 74 cells, 22 slices, 20 mice and *n* = 69 cells, 16 slices, 11 mice, respectively; processes 10.7 ± 0.7 vs. 22.2 ± 2.7, *P* < 0.0001, *n* = 91 processes, 22 slices, 20 mice and *n* = 76 processes, 16 slices, 11 mice, respectively; endfeet 7.2 ± 0.7 vs. 14.1 ± 2.1, *P* = 0.001, *n* = 36 endfeet, 22 slices, 20 mice vs. 14 endfeet, 16 slices, 11 mice).

At day 3 the Ca^2+^ signal amplitudes were still significantly elevated in astrocytic somata and endfeet, but not in processes (soma contralateral 6.8 ± 0.4 vs. ipsilateral 10.3 ± 0.8, *P* = 0.029, *n* = 74 cells, 22 slices, 20 mice and *n* = 39 cells, 6 slices, 3 mice respectively; processes 10.7 ± 0.7 vs. 16.6 ± 1.3, *P* = 0.12, *n* = 91 processes, 22 slices, 20 mice and *n* = 40 processes, 6 slices, 3 mice, respectively; endfeet 7.2 ± 0.7 vs. 18.4 ± 2.4, *P* < 0.0001, *n* = 36 endfeet, 22 slices, 20 mice vs. 9 endfeet, 6 slices, 3 mice, respectively). At day 7 the Ca^2+^ signal amplitudes in all astrocytic compartments had returned to control values. At day 1 post kainate injection the duration of the stimulation evoked Ca^2+^ signal was significantly increased in the astrocytic endfeet compared to the non-injected side (26.30 ± 2.8 vs. 14.83 ± 0.6, *P* < 0.0001, 20 endfeet, 16 slices, 11 mice, and *n* = 36 endfeet, 22 slices, 20 mice, respectively), while no changes were observed in other astrocytic processes or in the cell bodies. The latency from start of Scc stimulation to Ca^2+^ fluorescence increase was similar in all astrocytic subcompartments and not affected by kainate injection. However, kainate injection increased the Ca^2+^ signal rise rate in all compartments (for somata and processes at day 1; for endfeet at day 3; Figure 2B).

### The augmented stimulation evoked astrocytic Ca^2+^ responses following kainate injection was dependent on mGluR5

**Figure 3:**
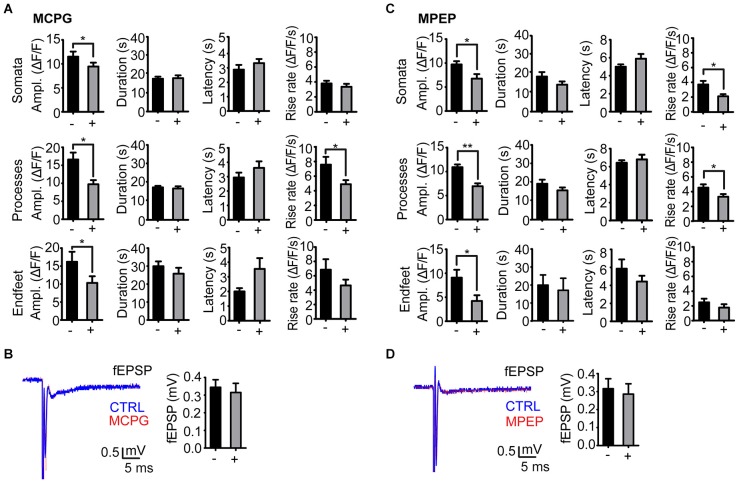
**Effect of mGluR antagonists on the stimulation evoked astrocytic Ca^2+^ signals in the kainate injected side. (A)** Amplitude, duration, latency and rise rate of Ca^2+^ signals in different astrocytic territories before (−) and after (+) administration of the mGluR I/II antagonist MCPG. **(B)** Representative fEPSP traces before (CTRL; blue) and after (red) MCPG administration and mean fEPSP amplitudes. **(C)** As in **(A)**, but with MPEP instead of MCPG. **(D)** As in **(B)**, but with MPEP instead of MCPG. Values are mean ± s.e.m. Asterisk, *P* < 0.05. Double asterisk, *P* < 0.0001.

Administration of the group I/II mGluR antagonist MCPG significantly reduced the amplitude of stimulation evoked Ca^2+^ signals in all astrocyte compartments at day 1 post kainate injection (soma, *P* = 0.04, 32 somata, 9 slices, 6 mice; processes *P* = 0.0001, 36 processes, 9 slices, 6 mice; endfeet *P* = 0.04, 14 endfeet, 9 slices, 6 mice). The duration and latency of the Ca^2+^ signals were not affected by MCPG, whilst rise rate was significantly reduced only in processes (Figure 3A).

As mGluR5 receptors have been shown to mediate enhanced astrocytic Ca^2+^ signaling following pilocarpine induced SE (Ding et al., 2007), we applied the mGluR5 selective antagonist MPEP in our model. Similarly to MCPG, administration of MPEP significantly reduced the amplitude of stimulation evoked Ca^2+^ signals at day 1 after kainate injection in astrocytic somata (*P* = 0.003, 19 somata, 5 slices, 4 mice), processes (*P* < 0.0001, 19 processes, 5 slices, 4 mice) and endfeet (*P* = 0.04, 7 endfeet, 5 slices, 4 mice). MPEP reduced the amplitudes of the Ca^2+^ transients by 30–40%, i.e., to the level at the non-injected side (Figure 3C). The nonselective antagonist MCPG reduced the Ca^2+^ transients to the same extent, suggesting that mGluR5 alone is mediating the enhanced Ca^2+^ signal amplitude in the latent phase. Similarly to MCPG, MPEP did not affect the duration and latency of the Ca^2+^ signals, and had inconsistent effects on transient rise rate in the three compartments (Figure 3C).

Neither MPEP nor MCPG significantly affected the fEPSP amplitudes (Figures 3B,D).

## Discussion

Astrocytes are highly polarized cells, structurally as well as functionally, opening for the possibility of a compartmentation of Ca^2+^ signaling analogous to that found in neurons. With the advent of genetically encoded Ca^2+^ sensors this possibility can be experimentally explored. A key question is whether Ca^2+^ signaling in the astrocytic endfeet could play a role in epileptogenesis, by initiating a sequence of events that lead to disassembly of the DAPC in the endfoot plasma membrane. This complex, known to be critical for K^+^ and water homeostasis in brain, is lost in patients with mesial temporal lobe epilepsy (Eid et al., 2005; Heuser et al., 2012) and in the latent phase of kainate induced epilepsy (Alvestad et al., 2013).

Here we show that intracortical kainate application leads to a stimulation evoked Ca^2+^ signal in the endfeet that outlasts the Ca^2+^ signal in the astrocytic cell bodies. This underlines the idea that endfeet are distinct subcompartments of astroglia (Nagelhus and Ottersen, 2013) and, more specifically, that endfeet serve as diffusion-limited subcellular compartments (Nuriya and Yasui, 2013).

The Ca^2+^ signal in endfeet is attenuated by blockade of group I/II mGluRs and thus dependent on Ca^2+^ mobilization from intracellular stores. However, mGluR blockade does not cancel out the difference between endfeet and cell bodies when it comes to the duration of the Ca^2+^ signal. This suggests that the increased signal duration primarily reflects reduced clearance of Ca^2+^. An uncoupling of astrocytes could contribute to reduced clearance (Bedner and Steinhäuser, 2013; Bedner et al., 2015).

A disassembly of the DAPC in astrocytic endfeet and the loss of astrocyte polarization that this entails now emerge as a signature event in mesial temporal lobe epilepsy and epilepsy models (Nagelhus and Ottersen, 2013). The disassembly is reflected by a loss of dystrophin DP71, while β-dystroglycan remains (Heuser et al., 2012). Beta-dystroglycan is a member of the DAPC and normally serves to link this complex to extracellular matrix molecules of the pericapillary basal lamina (Neely et al., 2001; Amiry-Moghaddam and Ottersen, 2003).

It has been proposed that the molecular disassembly in astrocytic endfeet is caused by calpain activation (Nagelhus and Ottersen, 2013). Calpain is capable of cleaving DP71, and the expression of this protease is increased in activated astrocytes (Shields et al., 2000). It has not been resolved, however, whether endfeet sustain Ca^2+^ signals necessary for activation of calpain or any other Ca^2+^ dependent protease with affinity for dystrophin or dystrophin associated molecules. The present study fills this void and shows that endfeet display Ca^2+^ signals that even outlast those in the astrocytic cell bodies. The cascade of events underlying the molecular disassembly in endfeet is an obvious target for future therapies.

## Author contributions

KS designed experiments, acquired, analyzed and interpreted data, and wrote the paper; KH conceived the study and supervised experiments, acquired, analyzed and interpreted data, and wrote the paper; WT designed experiments, acquired, analyzed and interpreted data, and wrote the paper; VJ designed and supervised experiments, interpreted data, and wrote the paper; RE analyzed and interpreted data, and wrote the paper; PB designed the animal model, interpreted data and commented upon the manuscript; CS designed the animal model, interpreted data and commented upon the manuscript; ET conceived the study, interpreted data and commented upon the manuscript; OPO conceived the study, interpreted data and wrote the manuscript; EAN conceived the study and supervised experiments, interpreted data and wrote the manuscript. All authors revised the work critically and approved the manuscript.

## Conflict of interest statement

The authors declare that the research was conducted in the absence of any commercial or financial relationships that could be construed as a potential conflict of interest.
